# A Qualitative Study Regarding COVID-19 Inpatient Family Caregivers’ Need for Supportive Care

**DOI:** 10.2174/1745017902117010161

**Published:** 2021-11-19

**Authors:** Angelo Picardi, Marco Miniotti, Paolo Leombruni, Antonella Gigantesco

**Affiliations:** 1 Centre of Behavioural Sciences and Mental Health, Italian National Institute of Health, Rome, Italy; 2 Department of Neuroscience, "Rita Levi Montalcini", University of Turin, Clinical Psychology Unit, AOU Città della Salute e della Scienza di Torino, Turin, Italy

**Keywords:** COVID-19, Family caregiver, Health professionals, Psychological impact, Focus group, Supportive care

## Abstract

**Background:**

Family caregivers of COVID-19 inpatients are exposed to multiple sources of distress. These include not only losing friends, colleagues and members of the family, but also the fear of possible losses in sociality, finances and, impoverished communication with sick family members and health care providers.

**Objective:**

This study describes the psychological experience of COVID-19 inpatient family caregivers to highlight the main sources of distress, issues, concerns and unmet needs.

**Methods:**

Two focus groups were independently organized with COVID-19 inpatient family caregivers and health care personnel of COVID-19 wards in order to highlight family caregivers’ practical and psychological burden and related needs. A thematic analysis was conducted to analyze the data.

**Results:**

Family caregivers mentioned they needed more information about the patient’s condition with more attention being paid to their own emotional state. Feelings of impotence, concerns about how to deal with patient’s discharge, significant psychological distress, and anxiety were frequently reported by study participants.

**Conclusion:**

Study findings suggest the need to strengthen the assistance of COVID-19 patient family caregivers. In the pandemic scenario, family caregivers might represent a crucial resource, which can guarantee rapid discharges, support home health care and thus relieve pressure on hospital systems.

## 1 INTRODUCTION

In August 2020, there were more than 20.5 million people infected by the 2019 novel coronavirus disease (COVID-19) worldwide and over 750,000 related deaths [1]. The resulting pandemic severely tested the public health and economic systems on a global scale. Apart from the damage to physical health caused by COVID-19, which has ranged from mild respiratory symptoms, to severe pneumonia, acute respiratory syndrome, septic shock and even systemic multiple organ failure syndrome, the need to stem the rapid spread of the disease inside hospitals, together with shortages of resources for testing and protecting, has led to the adoption of drastic measures concerning disinfection, social distancing, isolation, quarantine, which results in unfamiliar precautions in the management of COVID-19 inpatients and their family caregivers. These security measures, while needed to curtail infection spread, have considerably decreased the amount of psychological support that can be provided to patients, both by healthcare personnel, and, more seriously, by family caregivers. This lack has undoubtedly exacerbated the impact of psychological distress relating to uncertainties and fears about infection routes, disease treatment, course and long-term sequelae on COVID-19 inpatients’ mental health, with preliminary reports documenting the increased prevalence of anxiety and depression among this patient population [2-5].

Family members of COVID-19 inpatients had to accept that COVID-19 was becoming a leading cause of death in 2020. Therefore, for long periods, they are often confronted with the fear of losing a loved one, grieving and surviving in complicated and particularly distressing circumstances. Apart from all the other scares they shared with the general population in relation to the COVID-19 pandemic, they have to deal with one of life’s scariest fears, the death of a loved one, in a novel and highly undesirable situation. Thus, they are exposed to the threat of having multiple possible losses, not only in affections but also in sociality, work and finances. Many of those who had to face the loss of a family member were unable to say farewell to their loved one and often had also to deal with the impossibility of fulfilling ritual, social bereavement practices, such as the preparation of vigils, rosaries and funerals. Previous evidence has shown that deprivations and complications of these kinds increase the risk of developing prolonged grief reactions or disorders [6-8]. In addition, communication between health care providers and family caregivers has been poorly effective, impoverished and complicated by social distancing measures, which has also greatly reduced the opportunity to receive social support from other relatives or friends, who may also potentially be involved in similar issues and concerns. Therefore, the little evidence available to date seems to suggest that COVID-19 inpatient family caregivers represent a particularly vulnerable subpopulation at risk of complicated bereavement onset or of developing prolonged grief disorders [9, 10].

This study intends to address the experience, issues, concerns and needs for support of COVID-19 inpatient family caregivers, which is a topic that has not been extensively explored. To this purpose, a qualitative study using online/face-to-face focus groups and telephone interviews was carried out on small samples of COVID-19 inpatient family caregivers and health care personnel of COVID-19 wards.

## *2.* METHODS

### 2.1 Study Design, Participants and Procedures

This qualitative study was conducted at the Clinical Psychology Unit of the ‘Città della Salute e della Scienza di Torino’ University Hospital in April-May 2020. Two 60-90 minute focus groups were independently conducted with COVID-19 inpatient family caregivers (FG1) and health care personnel of COVID-19 wards (FG2). FG1 was moderated by a psychiatrist, while FG2 was moderated by a clinical psychologist. Also, a number of COVID-19 inpatient family caregivers who were unable to participate in a video meeting were contacted by telephone and underwent a semi-structured interview conducted by a clinical psychologist.

This study was approved by the Ethical Committee of the of the ‘Città della Salute e della Scienza di Torino’ University Hospital (protocol number 0039960) and it was conducted in accordance with the principles of the Declaration of Helsinki. Participants from both FG1 and FG2 were asked to give their consent regarding their participation in focus groups and permission to be audio-recorded. All study participants provided oral informed consent that was audio recorded.

### 2.2 Guidelines for focus Groups and Telephone Interviews

Focus groups and interviews explored answers and reflections concerning the following three main questions: 1) What are the information and communication needs of completely isolated COVID-19 inpatient family caregivers? 2) What is their practical burden? 3) What is their psychological burden? Tips used by moderators and interviewers to encourage debate are summarized in Table **1**.

### 2.3 Qualitative Analysis

The thematic analysis was chosen for this study. Audio recordings of focus group sessions and telephone interviews were transcribed in electronic form. The transcriptions were read several times to enable the authors to become familiar with the data. Initial topics were identified, noted, refined progressively and classified to identify themes and subthemes. Data saturation was considered to have been reached when no new themes or subthemes could be identified. The process of analysis was conducted according to Braun and Clarke’s thematic analysis approach [11]. The phases of this approach are: 1) transcribing, reading and rereading data, taking notes of initial ideas for coding; 2) generating initial codes (some initial codes may go on to form main themes, whereas others may form sub-themes, and others still may be discarded) and collating data relevant to each code; 3) searching for themes and sub-themes, *i.e.*, collating codes into potential themes, and considering how different codes may combine to form an overarching theme; 4) reviewing themes, *i.e.*, checking if the themes work in relation to the coded extracts, and generating a thematic map in which the themes fit well together; 5) defining and naming themes, *i.e.*, on-going analysing in order to refine the specificity of the themes; 6) producing a report of the analysis. To improve the reliability of this approach, two coders (AG, PL) independently classified material using this approach and only the themes on which the coders reached an agreement were included in the final report of the analysis.

## 3 RESULTS

As described in detail in Table **2**, FG1 was attended by three COVID-19 inpatient family caregivers (a daughter of a 74-year-old man, a sister of a 47-year-old man, a niece of an 83-year-old woman) and a volunteer assisting an 87-year-old widow without sons. Another four COVID-19 inpatient family caregivers (a daughter of a 79-year-old woman, a wife of a 69-year-old man, a wife of a 52-year-old man, a son of a 73-year-old patient) who were unable to participate in a video meeting underwent a semi-structured telephone interview. FG2 was attended by a dean of internal medicine, two medical directors from the ‘Città della Salute e della Scienza di Torino’ Direction, and two clinical psychologists.

As far as caregivers are concerned, we derived 4 major themes from qualitative analysis, which are described in detail in Table **2**: information needs, communication needs, practical burden, and psychological burden.

First, the caregivers expressed their need for frequent scheduled news about the patient during hospitalisation, and for receiving a complete post-discharge treatment plan taking into account comorbidities and previous therapies. In the event of a bad outcome, they expressed the need for reassurances that everything possible had been done for the patient and that he or she was not left alone at the time of death.

Second, some of them reported difficulties in communicating with the patient by telephone or video-call and gave suggestions to facilitate communication, such as equipping the COVID-19 wards with a wi-fi network accessible to patients, with cables for charging cell phone batteries, and with a person who could help the elderly use devices they cannot use on their own in order to communicate with family members. They expressed the need for proactive, regular communication with the healthcare staff, characterized by sensitivity and humanity.

Third, the caregivers described how they were facing a substantial practical burden, as they were facing several issues, such as difficult simultaneous remote management of the hospitalized patient and his or her cohabiting family members who remained at home, difficulty in shopping and finding medicines (especially if family members are quarantined), and extraordinary expenses for specialist visits, home assistance and paid ambulance transport.

Fourth, the caregivers reported substantial psychological burdens, including sleep difficulties, anxiety, feelings of guilt for neglecting family members other than the patient, and feelings of impotence for not being able to reassure the patient about the final outcome. They expressed the need for psychological support by phone, and for spiritual accompaniment.

Concerning health care personnel, we derived 4 major themes from qualitative analysis, which are described in detail in Table **3**: information and communication, practical burden, psychological burden, and treatment and care coordination.

First, they underscored the importance of providing families with regular updates and of using the same staff member for all communications. In case of a bad outcome, the professionals thought it important to agree with the family how many and which relatives would attend the last farewell, and to inform the family about the care given to the patient when the condition worsened irremediably and about the procedures used to prepare and preserve the corpse.

Second, they acknowledged that the families faced some issues even in the event of a positive outcome, once the patient has returned home, for instance, difficulties in retrieving objects the patient may need that were left in the hospital, and in obtaining sickness or hospitalization certificates for the employer.

Third, they underlined that hospital staff themselves carry a substantial psychological burden, as they may feel socially isolated, concerned for their own health, and helpless for not having helped or supported the patient enough.

Fourth, they recognised that there were some issues with treatment and care coordination and that the families faced a number of difficulties, for instance, in receiving remote psychological care and in maintaining contact with general practitioners.

The contents provided by both groups, which were mostly coherent with each other, were isolated, matched and integrated to identify the needs felt and demands asked by family caregivers (Tables **4** and **5**). The needs and relative demands are listed in Table **4**.

## 4 DISCUSSION

Learning from the challenges posed by the current pandemic is necessary in order to improve practices in the future. Understanding the needs of families and how they can be best met has substantial relevance for both clinical practice and health policy. The aim of this study was to explore the experience of COVID-19 inpatient family caregivers, trying to identify and highlight their issues, concerns, and support needs through focus group discussions and semi-structured phone interviews with patients’ family members and health care personnel.

Regarding communication and the need for information, the findings of this study show that COVID-19 patient family caregivers need targeted and empathic, proactive and clear, brief and periodic communication from health care personnel. In the event of severe worsening of the patient’s clinical conditions, they also desire to have accurate information about the prognosis, treatment and care provided, and, when death occurs, about the last moments of life and the process of dying. These results corroborate findings from cancer literature [12] and are consistent with observations across different types of inpatient care settings [13]. They are also in line with a recent qualitative study of relatives whose family member died during the COVID-19 pandemic, which highlighted the importance for relatives to be provided with clear and detailed information from health care professionals about their family member’s condition throughout the final weeks and days of life [14]. The findings of another recent qualitative study of bereaved family members of patients who died in hospital from severe COVID-19 showed that merely providing families with technical information is not enough, and that it is important for hospital personnel to put effort into establishing rapport and bonding with relatives in order to reduce their feelings of solitude and help them better understand the medical information [15].

The needs and relative demands for help and support reported by the family caregivers and reported by members of health care personnel that were interviewed regarding information and communication are in agreement with the five-step procedure required to facilitate a good communication in end-of-life care settings within the context of an intensive care unit proposed by Curtis and colleagues [16], and summarized in the mnemonic VALUE. This procedure is a reminder to value and appreciate what family caregivers say, to acknowledge their emotions, to help them in understanding the patient as a person, and to elicit questions from them.

As a rule of thumb, it is useful to provide empathic validations of fears and anxieties, which can reassure individuals that their reactions related to such an extreme event as the current pandemic are perfectly normal [17]. In addition, asking explorative questions to gauge the expectations of family caregivers about the patient’s disease and prognosis and encouraging the expression of emotions can help prevent misunderstandings with health care personnel.

Several proposals to guide clinical communication with patients and families dealing with COVID-19 have been put forward in literature during and after the first phase of the pandemic [18, 19]. These contributions try to provide phrases or words to choose/avoid when answering common questions or addressing frequent concerns experienced by family caregivers during restricted visits and virtual meetings. Moreover, providing written information can help strengthen and consolidate reassurances [20].

Furthermore, particular attention must be paid to the clarity and completeness of the information provided to family caregivers. When possible, consistency should be promoted in who contacts family members to help ensure continuity of care and timely communication with families [20, 21]. It is important to provide them with frequent updates on the clinical progress of the disease in order to align their expectations with reality and, if necessary, gradually prepare them for the possible death of their loved ones. Regarding this last point, in cases where the patient is in critical condition with a poor prognosis, study participants have also highlighted that particular attention must be paid by clinicians to obtain information on the patient’s values and preferences (*e.g*., whether or not to favor quality rather than duration of life) or choices concerning resuscitation procedures and end-of-life dispositions. Involving family caregivers in these discussions can reduce their level of anxiety and decrease the risk of depressive reactions after loss [22]. A helpful attitude in conducting these discussions can help maintain hope while at the same time preparing for the worst [20]. The proposal for conversations based on alternative scenarios in the face of life-threatening diseases highlights the links between the patient’s current condition, the events that may likely occur along the illness trajectory and the possible subsequent outcomes. This approach facilitates the visualization of possible scenarios in family caregivers’ minds and promotes their involvement in decision-making processes and planning [23]. A simple but effective example can be to discuss the best (*i.e.*, what will happen if all goes well) and the worst-case scenarios (*i.e.*, what will happen if all goes wrong). This protocol was effective in communicating with families of critically ill COVID-19 patients [24]. The importance of intensifying communication with family caregivers when the patient’s conditions become critical is also supported by other authors, who have recommended twice-daily contacts in cases of approaching death [25].

Regarding the need for help, study participants highlighted the need for psychological support in managing distress and challenges in returning to normalcy after patient’s discharge, and for spiritual support in facing issues and concerns related to death anxiety, grief and loss. These results are in agreement with previous findings among COVID-19 patient family caregivers [17]. It has been underscored that access to spiritual support is important for many families, whether or not they are religious, and that spiritual care during the pandemic should include helping relatives face and overcome fears and find meaning and hope, assisting when they express a need to confess or reconcile, attending to their existential suffering, addressing feelings of guilt and remorse, and offering death preparation assistance [20]. Apart from more or less structured and specialist psychological interventions (*e.g*., remote psychotherapy *via* Skype) and spiritual support provided by a priest to manage psychological and existential distress, some simple tricks in the supportive care routine can help alleviate the suffering of patients and family members and strengthen trust. Participants in this study suggested providing tips on relaxation techniques or self-help exercises (*e.g*., autogenic training, yoga) to manage mild to moderate anxiety, similarly to what has been reported in a previous study [26]. Providing brochures on how to learn simple breathing exercises (*e.g*., keeping a diary of breathing associated with relaxation techniques) or rules and good habits regarding how to rest and sleep well (*e.g*., sleeping regular hours, staying active, doing exercises, avoiding alcohol or heavy meals) can be useful to convey to family caregivers the sensation that they can do something to maintain their own wellbeing and to counteract their growing distress and anxiety.

Another relevant aspect highlighted by study participants that may contribute to the development of family caregivers’ feelings of impotence, anxiety and psychological distress regards the unmet need to feel actively involved in the patient’s care process when hospitalized. Obviously, this request is in stark contrast with the possibility for family caregivers to go to the hospital and visit their loved one in a pandemic context. Useful strategies to engage families in times of physical distancing include creating a system to have limited personal effects delivered to the patient’s room (*e.g*., children’s art or sports memorabilia), describing the patient’s environment to family members, including the presence of items sent from the family, and asking relatives about the patient’s favorite food, music, audiobooks, and television preferences in order to customize the patient’s environment [21]. Also, requesting more personal information from family caregivers by phone about the patient that is contained in his/her medical records can personalize the relationship with him or her. Alternatively, asking them *via* email or smartphone for a photo of the patient before the disease, or a favorite song or any other element or suggestion that can make the hospitalization more pleasant for the patient can represent opportunities to personalize care, to make family caregivers feel more involved and closer to the patient, and to help hospital staff members see who the patient was before becoming ill.

Certainly, maintaining direct, even though remote, contacts between the patient and family caregivers as much and long as possible is a crucial element. Participants in this study identified video calling as a preferred means of communication. Therefore, it is important to equip COVID-19 wards with devices and equipment that facilitate the chance to communicate in this way. Some COVID-19 wards considered in this study have equipped themselves with smartphones, tablets, chargers and power supplies. Nevertheless, some study participants reported difficulties in this regard. To overcome barriers to effective communication between the patient and his or her family, it has been recommended to facilitate delivery of communication devices from the family to the patient, and to provide free internet access to inpatients and assist them in connecting their devices [21]. Although paying attention to aspects such as these may seem alien to evidence-based medicine, in the opinion of the authors of this study and others [27], it is an element that should not be ignored in caring for patients and families during a devastating and long-lasting pandemic like the one we are experiencing. Indeed, the present situation calls for finding novel ways to maintain the connection between patients, relatives, and healthcare providers, with the aim of preserving the quantity and the quality of the information provided to the relatives and of addressing their needs for information and support [28].

## 5 LIMITATIONS

Due to the methodology of the qualitative research, the sample size of this study was limited and the study period was short. This limits the generalizability of the findings and the chance to observe progression across time, capture long-term sequelae and identify casual links. Moreover, due to the priority security measures in the current pandemic context, it was not possible to standardize data gathering procedures.

## CONCLUSION

This study provides a comprehensive understanding of the psychological experience of COVID-19 patient family caregivers. Areas of unmet needs were isolated and relative demands for further care and assistance were extracted. Family caregivers reported the need to receive more information about the patient’s condition and the need to calibrate the communication on their emotional state. The frustration of not being close and actively present in the care of the patient was a constant element. Study participants also reported significant psychological distress, anxiety and feelings of impotence, together with severe concerns about how to deal with patient’s discharge and return home. Overall, the study findings suggest further strengthening attention and assistance toward COVID-19 patient family caregivers.

Several studies [14, 15, 29, 30] have shown that the relatives of COVID-19 patients, especially those whose family members died [14, 15, 30], experience severe emotional distress and are among the most vulnerable social groups in the current pandemic. A screening assessment of the level of psychological distress and of risk factors for complicated bereavement of family members could help identify relatives who may be at particular risk for poor bereavement outcomes and mitigate the effects of the possible impending loss on their mental health [20]. Family caregivers with risk factors for complicated bereavement should be referred to services that can provide adequate follow-up and appropriate psychological interventions.

## Figures and Tables

**Table 1:** Tips to promote debate and reflections in focus groups and telephone interviews.

**Information and Communication**
• Information about the patient’s health status, treatment and prognosis
• Information about how to contact the patient and the health care personnel
• Information about how to plan treatment and care at home after discharge
• Information about hygiene and safety measures to be taken at home after discharge
• Clarity, completeness and frequency of the information
**Caregiver’s Practical Burden **
• Issues and problems in daily activities related to the patient’s illness (*e.g*., job loss, financial difficulties, lack of free time, limitations in social relations and in caring for other relatives)
• Issues and problems related to administrative practices (*e.g*., health certificate issuance, bonus requests, permits from the employer)
• Difficulty in decision making regarding the future in relation to the patient (*e.g*., financial, legal fields)
• Practical problems in caring for/ communicating with the patient (*e.g*., getting the patient what he/ she needs)
**Caregiver’s Psychological Burden**
• Psychological distress, severe concerns regarding the future, fears, persistent sadness, sleep disorders of family members or other relatives
• Need for help and psychological support
• Need for information and instructions about relaxation techniques and practices against anxiety (*e.g*., autogenetic training)
• Need for fast screening of psychological distress
• Need for remote psychological talks or psychotherapeutic sessions

**Table 2:** Characteristics of study participants and hospitalised family members.

Participant characteristic		Family member characteristic	
Sex	Relationship with the hospitalised family member	Sex	Age
Woman	Daughter	Man	74
Woman	Sister	Man	47
Woman	Niece	Woman	83
Man	Volunteer assisting a hospital patient	Man	87
Woman	Daughter	Woman	79
Woman	Wife	Man	69
Woman	Wife	Man	52
Man	Son	Man	73

**Table 3:** Themes and contents provided by family caregivers (FG1).

**Information**
Before patient admission in hospital
• Need for accurate non-contradictory information on hygiene and safety (*e.g*., how to use the oximeter, take body temperature, adopt kitchenware)
During hospitalization
• Need for information on the most suitable ways of obtaining news about the patient (*e.g*., telephone, smartphone, email)
• Opportunity to receive a summary sheet for contacts (*e.g*., telephone numbers, e-mails, web sites, names of personnel for reference)
• Need for frequent scheduled news about the patient (*e.g*., once a day, including doctor’s overall impression on patient’s general condition, physical status, mood and psychological response)
After discharge
• Need for information about where to find and how to use the oxygen cylinder, about oxygen dosages (if needed)
• Opportunity to receive a complete treatment plan taking into account comorbidities and previous therapies (*e.g*., ‘*My mother was told not to take the drugs she usually took. When she returned home she did not know if she should resume her therapies immediately or whether they could interfere with the drugs she had taken during hospitalization..*’
After death (where relevant)
• Need for reassurances that everything possible had been done for the patient and that he/she was not left alone at the time of death
**Communication **
Between patient and family caregivers
• Difficulty in communicating at a distance only, especially when the hospitalization is prolonged
• Difficulty or inability to use the telephone by elderly patients
• Inability to see the patient on video-call when he/she is no longer able to collaborate
Between family caregivers and healthcare personnel
• Need for a proactive, regular communication characterized by sensitivity and humanity
• Need for a phone number to find the referring doctor and receive a daily report about the patient, ask questions and obtain explanations
Proposal to facilitate communication
• Equip the COVID-19 wards with a wi-fi network accessible to patients
• Equip the COVID-19 wards with cables for charging cell phone batteries
• Equip the COVID-19 wards with a person that will help the elderly, especially those suffering from dementia, use devices they cannot use on their own so they can communicate with family members
• Provide the patient with familiar objects to give comfort and maintain intimate bonds with the family
**Practical Burden**
Assistance during hospitalization
• Difficult simultaneous remote management of the hospitalized patient and his/her cohabiting family members that remain at home
• Difficulty in getting the things to the patient (*e.g*., having information on hygiene and safety rules for the clean linen to be delivered to the patient)
Assistance before and after hospitalization
• Difficulty in obtaining COVID-19 tests for asymptomatic family members living with the patient
• Difficulty in getting COVID-19 elderly patients living alone to properly adhere to treatments
• Poor interaction between patient/ family members and the general practitioner (not in all cases)
• Poor integration between emergency personnel and the general practitioners
• Difficulty in organizing home assistance after discharge (*e.g.,* ‘*The interaction with the health care system was difficult regarding the planning of the treatment plan.. It was not clear who I should talk to.. And furthermore having to do everything by phone..*’)
Financial issues
• Extraordinary expenses for specialist visits, home assistance and paid ambulance transport
• Lost or reduced earnings due to time off work
Daily living
• Difficulty in shopping and finding medicines (especially if family members are quarantined)
• Difficulty in reorganizing coexistence within the family (*e.g*., do not leave the discharged patient or the deceased patient’s spouse alone)
• Take over the patient’s workload
• Increased domestic activities when family members are quarantined
**Psychological burden**
Distress and symptoms
• Sleep disorders
• Fear of having difficulty in concentrating
• Anxiety, worry, crying fits
• Despondency toward caring for the patient at home
• Feelings of guilt for neglecting family members other than the patient
Emotional difficulty in relating with the patient
• Not being able to reassure the patient about the final outcome
• Not being able to manage the patient’s fears, crying fits, feelings of hopelessness and death anxiety
Needs for supportive care
• Need for psychological support by phone
• Need for counselling about relaxation techniques (*e.g*., autogenic training, yoga exercises)
• Need for spiritual accompaniment

**Table 4:** Themes and contents provided by health care personnel (FG2).

**Information and communication**
About patient’s health status communicated to the family caregivers*
• Continuous complete, simple and understandable updates**
• Worsening of clinical conditions communicated gradually
• Using the same professional for all communications
About patient’s discharge
• Clarifying rules of thumb about social isolation or quarantine when the patient is discharged and/or one or more family members are positive for COVID-19
About patient’s decease
• What care was given to the patient when the condition worsened irremediably
• What procedures were used to prepare and preserve the corpse
• How many and which family members can attend the last farewell
• Reassuring about fears and doubts about loss or exchange of the corpses
**Practical burden**
Re-organization and administration in daily living‡
• Difficulty in resuming daily activities once the patient has returned home
• Difficulty in retrieving objects the patient may have need that were left in the hospital
• Difficulty in obtaining sickness/ hospitalization certificates for the employer
**Psychological burden**
Helplessness and sense of isolation
• Not having helped or supported the patient enough
• Not having controlled or prevented the infection
• Sense of social and work isolation
Concerns for their own health‡‡
• Anxiety, irritability, hyperactivity, sleep disorders
• Disease denial (their own and the patient)
**Treatment and care coordination**
Psychological care
• Family caregivers have difficulty in receiving remote psychological care
Coordination with primary care
• Difficulty in maintaining contacts with general practitioners (*e.g.,* ‘*As a physician, I would like to interact with patients and families’ general practitioners after discharge. There is a need for structured transfers from hospital to the assistance at home. General practitioners should act as case-managers and coordinate the transitions.*’)

*Notes*: *The communication with COVID-19 patient family members was managed by a doctor from the ‘Città della Salute e della Scienza di Torino’ University Hospital Health Department as a spokesperson for the ward doctors together with a clinical psychologist. The communication was conducted on the basis of a standardized format compiled by doctors and nurses who care for the patient. This approach was adopted with family members when the patient was hospitalized in COVID-19 or in Semi-intensive wards. When the patient was hospitalized in an Intensive Care Unit or in a Resuscitation ward, the communication was conducted by the anaesthetist, who offered the chance for psychological support when needed or desired. The communication took place by phone or *via* tablet and the hospital provided the devices for patients who did not have them.

**In particular, overall conditions, presence/absence of pneumonia, use of Continuous Positive Airway Pressure (CPAP) devices, pharmacotherapy, prognosis.

COVID-19 pandemic or worked at home in smart working.

‡‡When one or more family member is positive for COVID-19, some patients were discharged even when still positive themselves. Information on how and when to stop the quarantine were provided by the general practitioner.

**Table 5:** Summary of needs and demands of COVID-19 patient family caregivers.

**Information and Communication**
• Especially at first contact, a clinical psychologist should be present; prolonged psychological support is required • Communication by health care personnel should be periodic (once a day), proactive and conducted with humanity; gradually shorter intervals can be adopted to portend an imminent prognosis • Communication should be addressed to the same family member and conducted by the same personnel who is treating the patient (it would be desirable to have a phone number to call for emergencies) • Communication should be basically brief, simple and clear but above all appropriate to the family caregiver’s communicative register (some want more technical and in-depth information) • Communication channels should be agreed upon (some would like video calls; others, if the signs of illness and suffering are evident, prefer email communication) • It would be useful to have an operator in charge in the ward to help patients to get in touch with their family • It would be useful to receive information about hygiene (laundry) and safety (oximeter, oxygen cylinder) rules after patient’s discharge • In the event of decease, it is important to obtain information and reassurance that the patient has received adequate care, his/her wishes have been respected, his/her pain has been managed and was not left alone in his/her last moments • After decease, it is important to receive information about the procedures for preparing and storing the corpse and reassurance that the corpses have not been mixed up • When the decease is reported, it would be useful to be allowed the time to listen and offered psychological support (even for mourning)
**Practical burden**
Due to the difficulties encountered in the organization of home care before and after hospitalization, family caregivers would welcome: • Greater involvement of the primary care physician • Greater integration between hospital personnel and primary care physicians • Greater coherence about the need for hospitalization between emergency physicians, general practitioners, hospital personnel Those quarantined also would welcome help with grocery shopping, obtaining medicines or other necessities
**Psychological burden**
Family caregivers reported psychological suffering and distress and would welcome: • Screening for psychological distress or complicated grief • Remote (video/ phone call) targeted psychological support (or psychotherapy in the case of complicated grief) • Spiritual support
• Wellness and relaxation advice
